# Identification of a Small Interface between the Methyltransferase and RNA Polymerase of NS5 that is Essential for Zika Virus Replication

**DOI:** 10.1038/s41598-018-35511-3

**Published:** 2018-11-26

**Authors:** Timur Rusanov, Tatiana Kent, Mohsan Saeed, Trung M. Hoang, Crystal Thomas, Charles M. Rice, Richard T. Pomerantz

**Affiliations:** 1 Fels Institute for Cancer Research, Department of Medical Genetics and Molecular Biochemistry, Temple University Lewis Katz School of Medicine, Philadelphia, Pennsylvania USA; 2 Laboratory of Virology and Infectious Disease, Center for the Study of Hepatitis C, The Rockefeller University, New York, NY USA

## Abstract

The spread of Zika virus (ZIKV) has caused an international health emergency due to its ability to cause microcephaly in infants. Yet, our knowledge of how ZIKV replicates at the molecular level is limited. For example, how the non-structural protein 5 (NS5) performs replication, and in particular whether the N-terminal methytransferase (MTase) domain is essential for the function of the C-terminal RNA-dependent RNA polymerase (RdRp) remains unclear. In contrast to previous reports, we find that MTase is absolutely essential for all activities of RdRp *in vitro*. For instance, the MTase domain confers stability onto the RdRp elongation complex (EC) and and is required for *de novo* RNA synthesis and nucleotide incorporation by RdRp. Finally, structure function analyses identify key conserved residues at the MTase-RdRp interface that specifically activate RdRp elongation and are essential for ZIKV replication in Huh-7.5 cells. These data demonstrate the requirement for the MTase-RdRp interface in ZIKV replication and identify a specific site within this region as a potential site for therapeutic development.

## Introduction

Mosquito-borne Zika virus (ZIKV) is a member of the Flaviviridae family first identified in 1947 in the Zika Forest region in Uganda^1–3^. Although ZIKV infections have historically caused mild symptoms and can infrequently lead to Guillain-Barré syndrome, the recent massive spread of the virus to the Americas has caused the World Health Organization to declare an international health emergency^1–3^. This is due to the ability of the virus to cause a rare neurological condition in infants called microcephaly which is characterized by a significantly reduced head size and impaired neurological and motor skill development^4^. Strikingly, ZIKV is not only transmitted from mother to fetus, but also via sexual intercourse^1^. Recent evidence in mice also indicates that ZIKV infection in males may cause sterility^5^. Hence, our current knowledge of the detrimental effects of ZIKV infection appears to be limited.

ZIKV has spread to 60 countries including the United States where ZIKV infections have been reported in Florida and in Texas^6,7^. Although endeavors are underway to develop a vaccine, this can take several years and is not necessarily guaranteed to be effective. Thus, an alternative strategy to combat ZIKV, such as the development of anti-viral drug inhibitors, is an important area of research. Based on the past successes of using polymerase inhibitors to treat human immunodeficiency virus (HIV) and hepatitis C virus (HCV) infections, drug inhibitors of the ZIKV RNA-dependent RNA polymerase (RdRp) are likely to be similarly effective in treating ZIKV infections^8,9^. However, our current knowledge of how the ZIKV replication machinery functions is limited.

**Figure 1:**
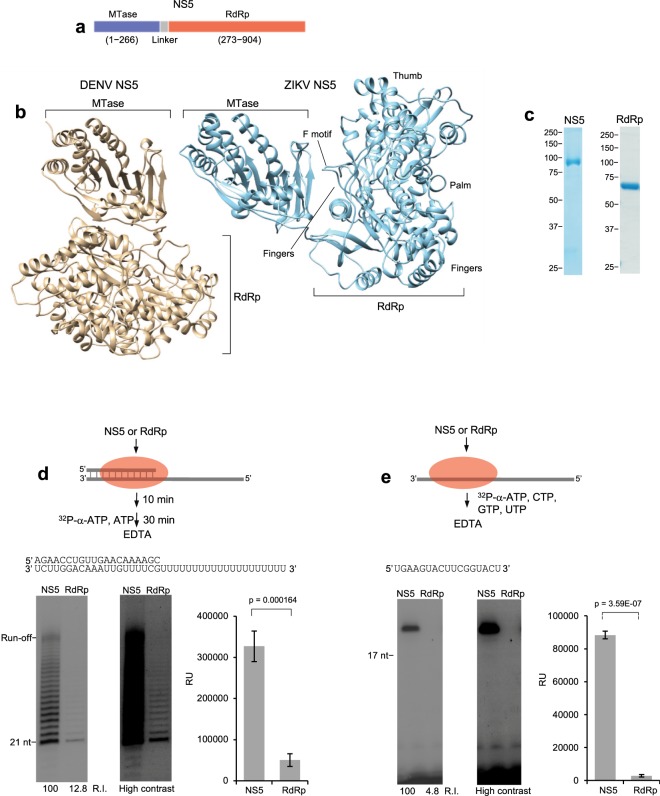
MTase is essential for RdRp elongation and initiation. (**a**) Schematic of ZIKV NS5 protein. (**b**) Comparison of ZIKV (right; PDB 5tfr) and DENV (left; PDB 4v0q) NS5 crystal structures highlighting different RdRp orientations. F motif, fingers, palm and thumb subdomains are indicated in ZIKV NS5 (right). (**c**) Denaturing SDS gels showing purified ZIKV NS5 (left) and RdRp (right). (**d**) Schematic of elongation assay (top). Denaturing gels showing elongation by RdRp and NS5 on indicated template (left). Bar chart showing the relative intensities of RdRp and NS5 elongation products generated after 30 min. *p* value determined by non-paired, two-tailed student’s *t*-test. n = 4, ± s.d. R.I. = relative intensity. RU = relative units (right). (**e**) Schematic of initiation assay (top). Denaturing gels showing *de novo* RNA synthesis by RdRp and NS5 on indicated template (left gels). Bar chart showing relative intensities of RdRp and NS5 *de novo* RNA synthesis products generated after 30 min. *p* value determined by non-paired, two-tailed student’s *t*-test. Data represent mean, n = 4, ±s.d. R.I. = relative intensity. RU = relative units (right). Uncropped and variable contrast gel images are presented in Supplementary Fig. 3.

All known Flavivirus RdRp enzymes are directly fused to a methytransferase (MTase) through a flexible linker within the non-structural 5 (NS5) protein that is essential for replication (Fig. 1a). The MTase performs 5′ RNA capping by facilitating guanine-N7 and nucleoside-2′-O methylation steps^10–12^. RNA capping promotes RNA stability, efficient translation and evasion of the host immune response^11,13^. Yet, why the MTase is directly fused to RdRp in the Flavivirus genus remains unclear. A plausible explanation is that these linked enzymes cooperate during RNA synthesis. Studies of related Dengue virus (DENV) report conflicting findings on the effects of the MTase on RdRp RNA synthesis activity. For example, stimulation and suppression of DENV RdRp by the MTase have been reported^14–16^, whereas another study showed that MTase had no effect on DENV RdRp activity^17^. Intriguingly, mutation of key residues involved in DENV MTase-RdRp interactions primarily resulted in an increase in RdRp initiation and elongation activities *in vitro*, suggesting MTase suppresses DENV RdRp activity^16^. A recent *in vitro* study using ZIKV proteins suggests that the MTase only significantly contributes to RdRp RNA elongation activity, which contrasts previous reports on DENV^14^. Clearly, detailed biochemical studies are required to unequivocally determine whether the effects of MTase on RdRp activity are universal among Flaviviruses or vary between different members such as ZIKV.

Recent structural studies reveal that the respective MTase domains of ZIKV and DENV NS5 lie in completely different orientations relative to RdRp (Fig. 1b)^18–21^. Japanese encephalitis virus (JEV) MTase exhibits the same conformation as ZIKV MTase^18–22^. A notable difference between ZIKV/JEV and DENV MTase conformations is their respective interactions with the RdRp fingers subdomain which is important for nucleotide binding and template interaction (Fig. 1b)^18,19,21^. Moreover, ZIKV/JEV MTase directly interacts with and stabilizes a motif in RdRp (motif F) which appears to contribute to the conformation of the NTP and template channels (Fig. 1b, right)^18–21^. In contrast, the F motif lacks interactions with MTase in DENV NS5, and thus is disordered^18^. Taken together, these studies suggest that the ZIKV/JEV MTase regulates RdRp by a distinct mechanism that may be critical for proper RdRp function in these viruses.

Here, we used biochemical and cellular approaches to understand how ZIKV MTase contributes to RdRp function during RNA replication. We unexpectedly found that the MTase domain of ZIKV NS5 is essential for the activity and stability of the RNA polymerase elongation complex (EC), and is also necessary for RNA initiation and nucleotide incorporation by RdRp. Structure function analyses identify key conserved residues in MTase that are important for RdRp elongation but not initiation. Importantly, when we mutated these residues to disrupt the specific MTase-RdRp interface site, ZIKV replication was completely abolished in Huh-7.5 cells. These data demonstrate that MTase acts as an essential auxiliary factor for ZIKV RdRp, and identify a small region within the MTase-RdRp interface as a potential drug binding site.

## Results

### MTase is essential for RdRp elongation and initiation

We initially used biochemistry to closely characterize ZIKV NS5 and RdRp RNA synthesis activities to determine the effects of MTase on RdRp mechanisms. The RdRp domain (274–904) encoded by the Asian ZIKV strain PRVABC59 was cloned into a *E*. *coli* expression vector containing a Hexa-histidine tag (His-tag) and a SUMO tag at the N-terminus. RdRp was purified by standard nickel affinity chromatography methods including cleavage of the His-SUMO tag (Fig. 1c, right). The RdRp domain from the Brazilian ZIKV strain SPH2015 was purified using similar methods, and controls show that the isolated RdRp domains from these respective strains exhibit nearly identical RNA elongation rates, and similar *de novo* RNA synthesis activities (Supplementary Fig. 1). Hence, these data demonstrate that strain specific variations within the RdRp domain do not significantly affect RNA synthesis activity, which is consistent with a prior report that mapped RdRp variations to the surface of the protein^18^. These data also show that purified RdRp exhibits consistent RNA synthesis activity for at least 40 min, which demonstrates that RdRp is stable and does not become inactivated during long incubation periods (Supplementary Fig. 1b). Full-length ZIKV NS5 from Asian strain PRVABC59 was purified by similar methods as RdRp with an additional gel filtration step (Fig. 1c, left).

We used similar RNA replication elongation assays performed previously with DENV and HCV RdRps^14,23^. For example, prior reports demonstrated the ability of DENV and HCV RdRps to extend synthetic RNA primer-templates in the presence of ribonucleoside triphosphates (NTPs)^14,23,24^. A multitude of studies on RNA polymerases, including those performed with HCV and DENV RdRps, show that extension of these types of primer-templates requires the polymerase to undergo a conformational change necessary for elongation of the RNA^23–27^. For example, recent structural studies of HCV RdRp demonstrate distinct initiation and elongation conformations of the enzyme, and many studies on DNA-dependent RNA polymerases show similar initiation to elongation conformational changes^25,27–32^. Reports also show the ability of DENV and ZIKV RdRps to readily conform to an EC on synthetic RNA primer templates, similar to DNA-dependent RNA polymerases^14,18,24^. In this report, we primarily focused on biochemical investigation of ZIKV RdRp and NS5 ECs.

RNA elongation was performed by pre-incubating NS5 or RdRp (both encoded by Asian strain PRVABC59) with a RNA primer-template at 37 °C for 10 min in order to allow the polymerase to adopt a conformation necessary for elongating the RNA, similar to previous studies (Fig. 1d, top)^33^. Next, ATP along with ^32^P-α-ATP were added and reactions were terminated after 30 min by the addition of EDTA and formamide. RNA was resolved in denaturing polyacrylamide gels and visualized by phosphorimager and autoradiography. The results show that NS5 is dramatically more proficient in RNA elongation activity compared to RdRp (Fig. 1d, left), and analysis of NS5 and RdRp RNA elongation under identical conditions in quadruplicate reveals a remarkable ~6 fold difference in elongation activity (Fig. 1d, right plot). A high contrast version of the elongation gel image demonstrates a similar but severely reduced product formed by RdRp compared to NS5 (Fig. 1d, right gel). Importantly, these reactions were performed on the same day using identical stocks of ^32^P-α-ATP, buffer and reaction components. Thus, the major difference in products generated by NS5 versus RdRp is exclusively due to their respective enzymatic activities, and not as a result of reaction artifacts or differences in reagents. These data provide new insight into the activity of RdRp. For example, recent studies highlighted the use of RdRp to investigate ZIKV replication *in vitro* and characterize potential inhibitors of the ZIKV replication machinery^34,35^. Yet, the data presented in Fig. 1d demonstrate that RdRp is severely defective in RNA elongation compared to NS5, which indicates that the MTase domain majorly contributes to RdRp activity.

Recent reports similarly found that RdRp is defective in RNA elongation, but found that the initiation activities of NS5 and RdRp were not significantly different^13^. We examined whether RdRp is impaired in RNA initiation activity compared to NS5 in Fig. 1e. Here, we used a RNA template lacking a primer to assess *de novo* RNA synthesis by the polymerase in the presence of NTPs and ^32^P-α-ATP (Fig. 1e, top). Again, the experiments were performed on the same day using identical stocks of RNA template, reagents and ^32^P-α-ATP to prevent any potential differences in reagent specific activity or integrity. In contrast to previous studies which used the identical RNA template to measure *de novo* RNA synthesis, we observed a remarkable ~31-fold reduction in *de novo* synthesis for RdRp compared to NS5 (Fig. 1e)^13^. We emphasize that the observed product is due to *de novo* RNA synthesis and not due to RNA 3′ terminal transferase of ^32^P-α-AMP. For example, the addition of ^32^P-α-AMP to the 3′ terminus of the unlabeled RNA would generate a product similar in length to that generated by *de novo* synthesis. However, we demonstrate as a control that under the identical reaction conditions, but using a 5′ radio-labeled RNA, NS5 is unable to transfer AMP to the 3′ terminus of the RNA substrate (Supplementary Fig. 2a). Remarkably, even after increasing the contrast of the gel image, RdRp initiation is barely detectable compared to NS5 (Fig. 1e, right gel). Because prior studies failed to observe a significant difference between NS5 and RdRp initiation activity^18^, our studies provide new insight into the effects of MTase on RdRp. For example, the results in Fig. 1 clearly show that MTase is essential for both elongation and initiation phases of RdRp RNA synthesis, which demonstrates that MTase acts as a necessary accessory factor for the polymerase during all phases of ZIKV replication.

### MTase confers stability onto RdRp

**Figure 2:**
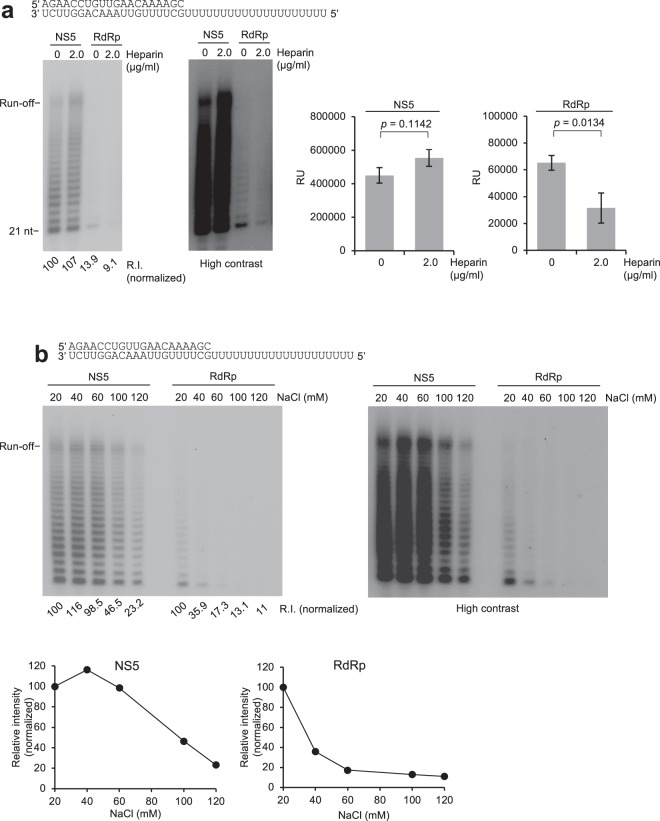
MTase is essential for the stability of the RdRp elongation complex. (**a**) Denaturing gels showing NS5 and RdRp RNA elongation for 30 min in the presence and absence of heparin (left). Bar charts showing relative intensities of NS5 and RdRp RNA elongation products with and without heparin (right). *p* value determined by paired, two-tailed student’s *t*-test. Data represent mean. n = 4, ± s.d. R.I. = relative intensity. RU = relative units (right). (**b**) Denaturing gels showing RNA elongation for 30 min by NS5 and RdRp in the presence of the indicated amounts of NaCl (top) R.I. = relative intensity. Scatter plots showing relative intensity normalized for each polymerase versus NaCl concentration (bottom). * = ^32^P. Uncropped and variable contrast gel images are presented in Supplementary Fig. 4.

Defective RNA elongation by RdRp could conceivably be due to more distributive activity compared to NS5. For example, because the MTase domain was previously shown to interact with the template via crosslinking^18^, it might contribute to the processivity of RNA elongation by helping to tether the RdRp domain to the template. To investigate this mode of action we tested whether NS5 was more resistant than RdRp to challenging agents during RNA elongation. First, we investigated the effects of heparin on the respective ECs. Heparin, a negatively charged polyanion, has been widely used to trap RNA polymerases after they dissociate from the template^36^. Remarkably, the NS5 EC is slightly stimulated by the presence of heparin, whereas the already impaired RdRp EC is further inhibited by heparin (Fig. 2a). A high contrast version of the gel emphasizes the inhibitory effect of heparin on RdRp elongation activity which is quantitated in the right plot (Fig. 2a, right gel). Hence, these data suggest that the NS5 EC is more stable than the RdRp EC.

We further compared the stability of the respective ECs by challenging them with increasing concentrations of NaCl (Fig. 2b). Here, once the halted ECs were formed, the RNA elongation was initiated by the addition of NTPs with increasing amounts of NaCl. Because nucleic acid polymerases interact with the phoshphate backbone of their respective substrates via multiple ionic interactions, unstable polymerase-nucleic acid complexes can be dissociated by relatively low amounts of NaCl. The results show that the RdRp EC is almost completely inactivated upon exposure to 60 mM NaCl, whereas the NS5 EC is highly resistant to the same amount of salt and requires at least 120 mM NaCl to become destabilized (Fig. 2b). Hence, these data indicate that the NS5 EC is substantially more stable than the RdRp EC as shown by its higher resistance to challenging agents heparin and NaCl.

### RdRp and NS5 exhibit similar affinities for template RNA

**Figure 3:**
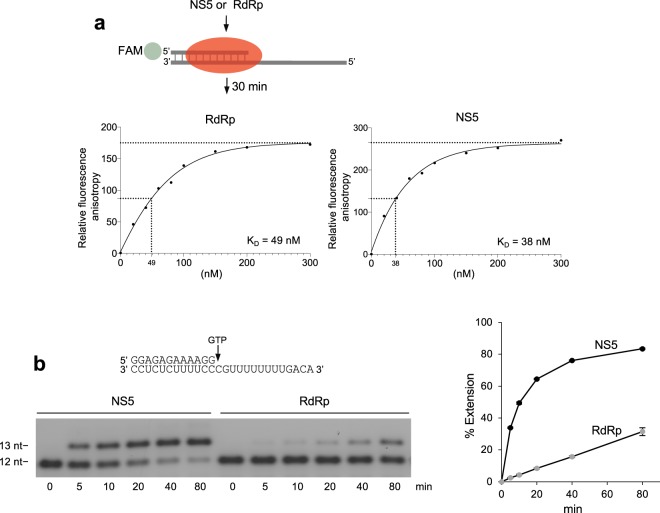
MTase facilitates nucleotide incorporation by RdRp. (**a**) Schematic of fluorescence anisotropy assay (top). Scatter plots showing relative fluorescence anisotropy for NS5 and RdRp template binding (bottom). Data represent mean, n = 3 ± s.d. (**b**) Denaturing gel showing time course of single nucleotide incorporation by NS5 and RdRp on the indicated elongation template. Scatter plot showing % extension over time by NS5 and RdRp (right). Data represent mean. n = 3, ± s.d. Uncropped gel images are presented in Supplementary Fig. 5.

Because NS5 is more stable than RdRp during the elongation phase, we investigated whether NS5 binds the primer-template with higher affinity. Here, we used a fluorescence anisotropy assay to measure the respective equilibrium dissociation constants (K_D_) of NS5 and RdRp (Fig. 3a). Unexpectedly, the results showed that the K_D_ of NS5 is only slightly lower than that of RdRp, demonstrating that their respective affinities for the primer template are similar. Hence, the defective elongation activity exhibited by RdRp is not likely due to a lower affinity for the template.

### MTase contributes to the rate of RdRp nucleotide incorporation

Another mechanism by which MTase may significantly contribute to RdRp activity is via catalytic activity. Recent structures demonstrate that MTase directly interacts with the F motif within RdRp which appears to contribute to the NTP binding channel conformation^18,19^. We therefore compared the relative velocity of a single nucleotide incorporation event by NS5 and RdRp. These reactions were performed on the same day using identical template and reagents to prevent possible discrepancies in conditions. Remarkably, despite their similar affinities for the RNA primer-template (see Fig. 3a), our results demonstrate that NS5 exhibits a dramatically higher relative rate of nucleotide incorporation compared to RdRp (Fig. 3b). The plot at the right quantitates these primer extension reactions performed in triplicate. Importantly, these data also demonstrate that RdRp is fully active even after 80 min of incubation which shows that the RdRp protein construct is not unstable. These findings indicate that MTase greatly contributes to nucleotide binding and/or catalytic activity, presumably by promoting proper configuration of the NTP binding channel.

### Identification of MTase residues involved in RdRp elongation

**Figure 4:**
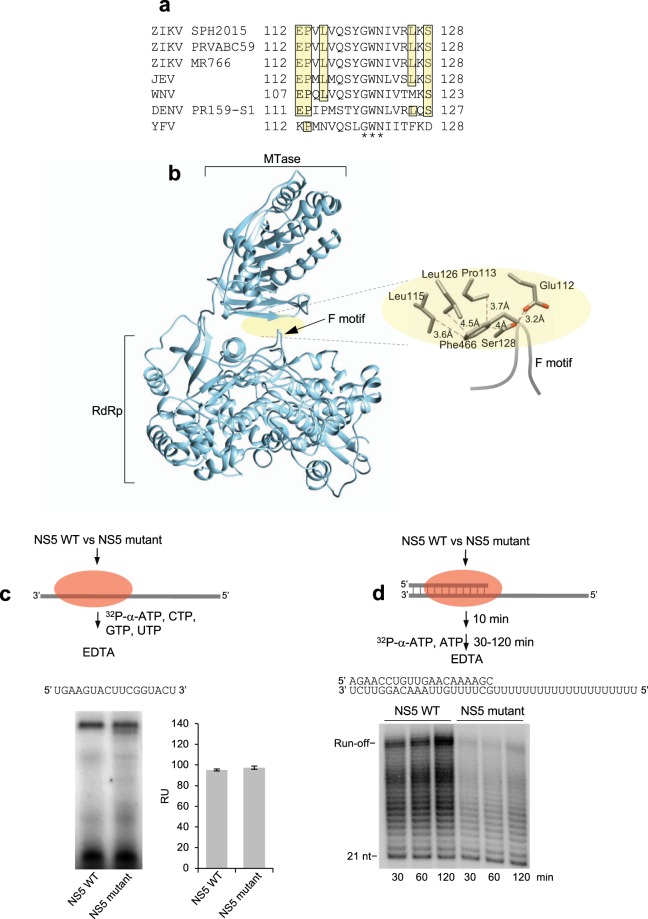
Identification of MTase residues that contribute to RdRp elongation activity. (**a**) Alignment of MTase residues that interact with the ZIKV RdRp F-motif. Highlighted residues directly contact tip of F motif. * = highly conserved residues in Flaviviruses. (**b**) Crystal structure of ZIKV NS5 (PDB 5tfr) highlighting conserved ZIKV/JEV MTase-F motif interactions. (**c**) Schematic of initiation assay (top). Denaturing gel showing *de novo* synthesis for 30 min by NS5 wild-type and mutant proteins on indicated template with 2 µM NTPs (left). Bar chart showing average *de novo* synthesis products generated after 30 min by NS5 wild-type and NS5 mutant enzymes. Data represent mean, n = 3, ± s.d. RU = relative units (right). (**d**) Schematic of elongation assay (top). Denaturing gel showing time course of elongation by NS5 wild-type and mutant proteins on indicated template (bottom). Uncropped and gel images are presented in Supplementary Fig. 5.

Since MTase interacts with multiple motifs in RdRp, it is difficult to predict which contacts are important for regulating RdRp activity. Nevertheless, we decided to identify specific MTase-RdRp interactions that contribute to the function of the polymerase. Based on recent structures of ZIKV NS5^18,19^, we chose to investigate conserved ZIKV/JEV MTase residues that directly interact with the fingers subdomain of RdRp (Fig. 4a). We focused on MTase residues E112, P113 and L115 that appear to stabilize the F motif in ZIKV NS5 which likely contributes to substrate channel folding based on structural analysis (Fig. 4b)^18,19^. For example, in ZIKV NS5 the tip of this motif involving conserved F466 directly contacts residues E112, P113 and L115 via hydrophobic and ionic interactions (Fig. 4b). In RdRp, however, the F motif lacks these contacts and lies in a completely different orientation that appears to interfere with the NTP channel in crystal structures^18^. Importantly, these specific MTase-RdRp interactions are observed in ZIKV/JEV, but not in DENV. Furthermore, these residues highlighted in Fig. 4a are not absolutely conserved among Flavivirus NS5, suggesting unique functional contacts in ZIKV/JEV.

We mutated residues E112, P113 and L115 to glycine to prevent alternative intra- or inter- domain interactions that would be difficult to interpret without structural information. Elongation and initiation assays were then employed to directly compare the respective RNA synthesis activities of wild-type and triple mutant NS5. Remarkably, we find that the triple mutant enzyme lacking these key MTase-RdRp interactions is deficient in elongation activity (Fig. 4d), but is not impaired in initiation even at relatively low (2 µM) NTP concentrations (Fig. 4c). We note, however, that the triple mutant is slightly deficient in synthesizing a run-off product during the initiation assay (Fig. 4c) as indicated by the top double band, and this is also observed at a 5-fold higher concentration of NTPs (Supplementary Fig. 2b). Taken together, the data presented in Fig. 4 map MTase-RdRp interactions that strongly contribute to RNA elongation but not *de novo* RNA synthesis, and thus demonstrate a direct but non-enzymatic function for MTase in regulating the NS5 EC.

**Figure 5:**
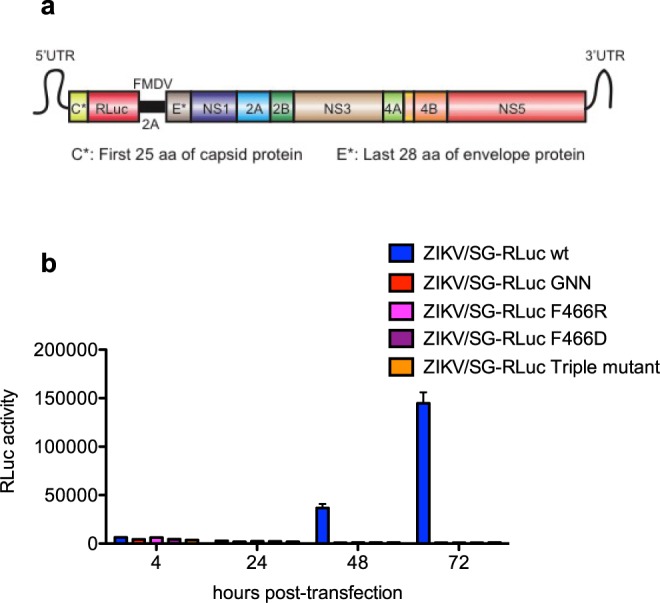
The MTase-RdRp interface is essential for ZIKV replication in cells. (**a**) Schematic of ZIKV replicon (ZIKV/SG-RLuc). (**b**) Huh-7.5 cells were transfected with the indicated replicons, and luciferase activity was measured at 4, 24, 48, and 72 hours post-transfection. Data represent mean, n = 4, ± s.d.

Lastly, we tested the relevance of these residues for cellular ZIKV replication. First, we generated ZIKV subgenomic replicons by replacing the viral genomic region encoding parts of the structural proteins (from amino acid 26 of the capsid protein through amino acid 476 of the envelope protein) with a cassette containing the Renilla luciferase followed by the foot-and-mouth disease virus (FMDV) 2 A protease. The resulting ZIKV replicon (ZIKV/SG-RLuc wt) contains the 5′UTR, the first 25 amino acids of the capsid protein, Renilla luciferase, FMDV 2 A protease, the last 28 amino acids of the envelope protein, viral NS1-NS5 proteins, and the 3′ UTR (Fig. 5a). The results show that the replicon replicates to high levels in Huh-7.5 human hepatoma cells (Fig. 5b). However, when the MTase-RdRp interface was disrupted by mutating key MTase residues E112, P113 and L115 (triple mutant), or the opposing RdRp residue F466 (F466D or F466R), replication was completely abrogated as indicated by a lack of luciferase activity (Fig. 5b). As a control, we show that an NS5 RdRp active site mutant (GNN; residues 663–665) exhibits a similar defect in replication as expected (Fig. 5b). Taken together, these cellular studies confirm an essential function for the MTase-RdRp interface in ZIKV replication and identify a specific site between the F motif and MTase as a potential region for therapeutic development.

## Discussion

Because viral polymerases, such as those encoded by HIV and HCV, have demonstrated to be highly effective drug targets, the RdRp domain of ZIKV NS5 is similarly considered a promising enzyme for therapeutic development^8^. Thus, understanding how the ZIKV NS5 RdRp domain functions and is regulated at the molecular level is important for developing therapeutics against its enzymatic activity. A previous study has reported that the MTase-RdRp interface in ZIKV NS5 only significantly affects elongation activity^18^. In contrast to this previous report, our studies demonstrate that the MTase domain is essential for all RNA synthesis activities of RdRp. For example, we demonstrate that RdRp is severely deficient in *de novo* RNA synthesis, EC stability, and nucleotide incorporation. Although multiple reports have specifically focused on the activity of the isolated RdRp domain^35,37,38^, our findings show that it is severely defective in RNA elongation and initiation.

Our studies identify a small MTase-RdRp interaction site that is essential for ZIKV replication in cells. Biochemical data of the identical NS5 mutant *in vitro* demonstrates that this interface is important for RNA elongation. Hence, our findings indicate that the specific MTase-RdRp interface formed by residues E112, P113 and L115 of the MTase domain with RdRp F466 is essential for ZIKV replication by promoting replication elongation. The possibility exists that these residues potentially interact with other ZIKV proteins during the course of replication and thus could conceivably have additional undefined roles in replication, in addition to their replication elongation function.

Importantly, recent crystallographic studies complement our data and provide further insight into potential mechanisms by which the MTase regulates RdRp. For instance, structural data demonstrate that the MTase contributes to the conformation of the NTP and template binding channels^18,19^. Specifically, MTase residues that stabilize the F motif (illustrated in Fig. 4b) were shown to bind the template via crosslinking experiments^18^. Thus, in addition to interacting with and regulating the RdRp fingers subdomain, the MTase also functions in template binding. This may explain why the RdRp EC is much less stable than the NS5 EC in our studies. Motif F in the isolated RdRp domain crystal lacks functionally important MTase contacts, such as residues E112, P113, and L115^18,19^. In the absence of these and other stabilizing MTase contacts (i.e. L126), the F motif structure is dramatically changed and as a result alters the configuration of the NTP and template binding channels^18,19^. This structural alteration likely explains why RdRp incorporates nucleotides at an extremely low rate compared to NS5 during elongation. Consistent with our biochemical observations, we find that identical MTase-F motif contacts in NS5 are essential for ZIKV replication in cellular studies, and these contacts are also needed for JEV replication, demonstrating a conserved function at least between ZIKV and JEV^39^.

Several studies have focused on inhibiting the respective active sites of RdRp or MTase^33,34,40–46^. Our findings indicate that the MTase-F motif interactions in ZIKV NS5 represent a potential site for therapeutic development. For example, compounds designed to specifically prevent the MTase-F466 interaction by binding key contacts in the 112–126 residue region in MTase (see Fig. 4b) are likely to inhibit elongation by the ZIKV replication machinery based on our biochemical and cell data. Altogether, our report in conjunction with recent structural studies demonstrate that the MTase domain is essential for proper ZIKV RdRp structure and function, and demonstrate that full-length ZIKV NS5 should be used to develop inhibitors of the polymerase domain.

## Experimental Methods

### RNA elongation

50 nM of the indicated RNA template (467 R/475 R) was pre-incubated with 1 µM or indicated concentrations of RdRp or NS5 proteins for 10 min in the following buffer (40 mM TrisHCl, pH 6.8, 10% glycerol, 2 mM MnCl_2_, 0.1 mg/ml BSA, 0.01% NP-40, 2 mM DTT) at 37 °C. RNA elongation was then initiated by the addition of 2 µM ATP along with 0.5 µl of ^32^P-α-ATP (Perkin Elmer) in a final volume of 20 µl. Reactions were terminated after 30 minutes by the addition of 25 mM EDTA and 45% formamide. RNA was then resolved in denaturing urea polyacrylamide gels containing 15% formamide, then visualized by autoradiography or phosphorimager. In Fig. 3b, 100 nM radio-labeled 456/457 and 300 µM GTP were used and reactions were terminated at indicated time intervals. Percent extension was determined by dividing the intensity of the upper band by the sum of the intensities of the upper and lower bands. Final NaCl concentration in elongation reactions from addition of protein and DNA storage buffers was 20 mM except where indicated. Relative intensity (R.I.) was determined by measuring the intensities of RNA elongation or initiation products using ImageJ for each set of experiments performed on the same day using identical reagents and radio-labeled nucleotides, then dividing the lower intensity value by the higher intensity value to generate % intensity of the lower value. The higher intensity value was then normalized to 100, and the relative intensity of the lower value was calculated by multiplying 100 by the % intensity of the lower value.

### Terminal transferase activity assay

50 nM of the ^32^P-labeled RNA substrate (478 R) was preincubated for 30 s with 1 µM of NS5 or RdRp in the following buffer (40 mM Tris-HCl pH 6.8, 2 mM DTT, 0.1% NP-40, 0.1 mg/ml BSA, 10% glycerol, 2 mM MnCl_2_) at 37 °C. Then 200 µM of NTPs were added for 1 hour until the reactions were terminated by the addition of 25 mM EDTA and 45% formamide. RNA was resolved in denaturing urea polyacrylamide gels and visualized by phosphorimager and autoradiography.

### *De novo* RNA synthesis

50 nM of the indicated RNA (RP478R) was pre-incubated with 1 µM NS5 or RdRp for 30 s in the following buffer (40 mM Tris-HCl pH 6.8, 2 mM DTT, 0.01% NP-40, 0.1 mg/ml BSA, 10% glycerol, 2 mM MnCl_2_). Then 10 µM or 2 µM of NTPs were added along with ^32^P-α-ATP (Perkin Elmer) for the indicated times. Reactions were terminated by the addition of 25 mM EDTA and 45% formamide and RNA was resolved in denaturing urea polyacrylamide gels and visualized by phosphorimager and autoradiography. Relative extension (R.E.) was determined by comparing the relative intensities of run-off products for each experiment and normalizing the most intense run-off product band to 100.

### RNA elongation template assembly

RNA primer was mixed with a 1.25 fold excess of template RNA in the presence of 50 mM NaCl, then the mixtures were heated to 70–80 °C for 2–5 min, followed by slow cooling to room temp. In the case of Figs 2b and 3b, and (Supplementary Figs 1b and 2a) the RNA primer was 5′ ^32^P labeled using T4 bacteriophage polynucleotide kinase (New England Biolabs) in the presence of ^32^P-γ-ATP (Perkin Elmer).

### Fluorescence anisotropy

Fluorescence anisotropy was performed by incubating the indicated amounts of NS5 or RdRp with 10 nM of fluorescein (FAM) conjugated RNA template (RP467RFAM/RP475R) at room temp in the following buffer: 20 mM Tris-HCl pH 6.8, 0.1 mg/ml bovine serum albumin (BSA), 0.01% NP-40, 10 mM dithiothreitol (DTT), 10% glycerol. After 30 minutes, florescence polarization of FAM was determined using a Clariostar plate reader. K_D_ was determined from protein concentration giving half-maximal template binding.

### Protein purification

ZIKV RdRp purification. ZIKV RdRp amino acid sequence 274–904 (Brazilian strain SPH2015) was cloned into pE-SUMOstar Amp vector *E*. *coli* vector (LifeSensors) resulting in a N-terminal Hexahistidine-SUMO tag. RdRp was expressed overnight at room temp in Rosetta2(DE3)/pLysS cells (Stratagene) after reaching 0.6 optical density (O.D.) and adding 0.4 mM IPTG. Cells were lysed by sonication in the presence of lysis buffer (25 mM TrisHCl pH 7.5, 500 mM NaCl, 10% glycerol, 0.005% IGEPAL, 2 mM DTT, 10 mM imidazole) and protein extract collected after ultracentrifugation at 35,000 rpm for 45 min. Clarified extract was loaded onto a His-trap crude 5 ml column (GE Healthcare Life Sciences), washed with 15 column volumes of lysis buffer. Protein was then eluted with lysis buffer containing a 10–250 mM imidazole gradient. Peak fractions containing His-SUMO RdRp were pooled and dialyzed into lysis buffer containing 200 mM NaCl and 0 imidazole, then mixed with SUMOStar protease (LifeSensors) and incubated overnight at 4 °C. Finally, cleaved RdRp was purified over a His-trap 5 ml column with a shallow gradient of imidazole. Pure cleaved RdRp was concentrated to 9.11 mg/ml and stored in aliquots at −80 °C. All steps in the purification process were carried out at 4 °C. RdRp encoded by strain SPH2015 was used in Figs 1 and 2, 3a, and Supplementary Fig. 1 where indicated. RdRp encoded by Asian strain PRVABC59 was purified in a similar manner to RdRp from Brazilian strain SPH2015 with the following modifications. The dialysis buffer used for SUMO cleavage used 50 mM TrisHCl pH 8.8. Pure cleaved protein was dialyzed against storage buffer containing 50 mM Tris 8.8, 350 mM NaCl, 10% Glycerol, 0.05% Igepal. RdRp encoded by Asian strain PRVABC59 was used in Fig. 3b and Supplementary Fig. 1 as indicated. NS5 purification. pE-SUMOstar Amp vector (LifeSensors) containing wild-type NS5 (Asian strain PRVABC59) was transformed into Rosetta2(DE3)/pLysS cells (Stratagene). Freshly grown colony was resuspended in 20 ml LB broth and incubated overnight at 37 °C. 1 ml of overnight culture was added to 1 L of autoinduction medium (1X Terrific Broth (USB Corporation), 0.5% w/v glycerol, 0.05% w/v dextrose, 0.2% w/v alpha-lactose, 100 μg/ml ampicillin and 34 μg/ml chloramphenicol) in a 2.8 L Fernbach flask. The flasks were shaken at 16 °C for 100 hours. 6 L of culture were grown and resulting *E*. *coli* pellets were stored at −80 °C. Frozen pellets were thawed on ice and resuspended in buffer containing 50 mM TrisHCl pH 8.8, 500 mM NaCl, 10% (v/v) glycerol, 10 mM imidazole pH 8, 1.5% (v/v) Igepal CA-630 (Sigma), 5 mM 2-mercaptoethanol (BME), 10 mM PMSF, and 1 SIGMAFAST™ Protease Inhibitor tablet per every 100 ml at a volume of 10 ml of buffer per gram of cell pellet. The resuspended cells were sonicated on ice and then centrifuged at 40,000 g. The clarified cell lysate was loaded onto a 5 ml HisTrap FF Crude column (GE Lifesciences) and washed with buffer A (50 mM Tris pH 8.8, 300 mM NaCl, 10% (v/v) glycerol, 10 mM imidazole pH 8.0, 5 mM BME and 0.005% v/v Igepal CA-630). Bound fractions were then eluted with a gradient to buffer B (50 mM Tris pH 8.8, 300 mM NaCl, 10% (v/v) glycerol, 0.005% (v/v) Igepal CA-630, 5 mM BME and 250 mM imidazole pH 8.0). Fractions containing NS5 were pooled, mixed with 10 units of SUMOStar protease (LifeSensors) and dialyzed against buffer C (50 mM Tris pH 8.8, 450 mM NaCl, 10% (v/v) glycerol, 5 mM DTT and 0.005% v/v Igepal CA-630) for overnight at 4 °C. The digested fractions were then loaded onto a 5 ml HisTrap HP column and washed with buffer C. Cleaved NS5 was separated from uncleaved protein and the protease by applying a gradient to buffer B. Fractions containing NS5 were concentrated, and loaded onto gel filtration column Superdex 200 Increase 10/300 GL (GE Healthcare) and run in the buffer 50 mM TrisHCl pH 8.8, 500 mM NaCl, 10% glycerol, 0.001% Igepal, 10 mM DTT. NS5 fractions were concentrated, frozen in 5 μl aliquots and stored at −80 °C. All steps in the purification process were carried out at 4 °C. NS5 triple mutant SUMOstar expression plasmid was generated using QuikChange II XL Site-Directed Mutagenesis Kit (Agilent Technologies). The NS5 triple mutant protein was purified in an identical manner to wild-type NS5.

### Construction of ZIKV replicons

The infectious clone of ZIKV Cambodian strain (FSS13025) is published^47^. It contains the viral genome flanked by a T7 promoter and hepatitis delta virus ribozyme sequence (HDVr) at the 5′ and 3′ ends, respectively. To generate the ZIKV subgenomic replicons, we replaced the region of the viral genome encoding amino acid 26 of the capsid protein through amino acid 476 of the envelope protein with Renilla luciferase (RLuc) followed by the foot-and-mouth disease virus (FMDV) 2 A protease. The resulting replicon, ZIKV/SG-RLuc, contains the 5′UTR, first 25 amino acids of the capsid protein, Renilla luciferase, FMDV 2 A protease, last 28 amino acids of the envelope protein, viral NS1-NS5 proteins, and the 3′ UTR. We generated the replication-defective replicon by mutating the RdRp active site motif GDD (NS5 residues 663–665) to GNN. Mutations in the ZIKV polymerase and methyltransferase domains were introduced by overlapping PCRs.

### Measurement of ZIKV replication in cells

Huh-7.5 cells were seeded into 24-well plates at the density of 50,000 cells per well. Twelve hours later, the cells were transfected with 125 ng of *in vitro* transcribed RNA from ZIKV replicons using TransIT-mRNA Transfection kit (Mirus, WI). The cells were harvested at 4, 24, 48, and 72 hours post-transfection using 1X cell culture lysis reagent (Promega, WI), and the Renilla luciferase activity was measured using FLUOstar Omega (BMD Labtech, Germany).

## Electronic supplementary material

Supplementary Figures

## References

[1] van de Beek Diederik, Brouwer Matthijs C. (2017). 2016, the year of Zika virus. Nature Reviews Neurology.

[2] Faria, N. R. *et al*. Zika virus in the Americas: Early epidemiological and genetic findings. *Science (80-*.*)*. **352** (2016). 10.1126/science.aaf5036 PMC4918795 27013429

[3] Lessler J., Chaisson L. H., Kucirka L. M., Bi Q., Grantz K., Salje H., Carcelen A. C., Ott C. T., Sheffield J. S., Ferguson N. M., Cummings D. A. T., Metcalf C. J. E., Rodriguez-Barraquer I. (2016). Assessing the global threat from Zika virus. Science.

[4] Li H, Saucedo-Cuevas L, Shresta S, Gleeson JG (2016). The Neurobiology of Zika Virus. Neuron.

[5] Ma W (2016). Zika Virus Causes Testis Damage and Leads to Male Infertility in Mice. Cell.

[6] Ventura CV, Albini TA, Berrocal AM (2016). First Locally Transmitted Zika Virus Cases Identified in the United States. JAMA Ophthalmol..

[7] Deckard DT (2016). Male-to-Male Sexual Transmission of Zika Virus — Texas, January 2016. *MMWR*. Morb. Mortal. Wkly. Rep..

[8] Jordheim LP, Durantel D, Zoulim F, Dumontet C (2013). Advances in the development of nucleoside and nucleotide analogues for cancer and viral diseases. Nat. Rev. Drug Discov..

[9] Mehellou Y, Rattan HS, Balzarini J (2018). The ProTide Prodrug Technology: From the Concept to the Clinic. J. Med. Chem..

[10] Chatrin C, Talapatra SK, Canard B, Kozielski F (2018). The structure of the binary methyltransferase-SAH complex from Zika virus reveals a novel conformation for the mechanism of mRNA capping. Oncotarget.

[11] Coloma J, Jain R, Rajashankar KR, García-Sastre A, Aggarwal AK (2016). Structures of NS5 Methyltransferase from Zika Virus. Cell Rep..

[12] Coutard, B. *et al*. Zika Virus Methyltransferase: Structure and Functions for Drug Design Perspectives. *J*. *Virol*. **91** (2017). 10.1128/JVI.02202-16 PMC5309936 28031359

[13] Dong H (2010). Biochemical and genetic characterization of dengue virus methyltransferase. Virology.

[14] Potisopon, S. *et al*. The methyltransferase domain of dengue virus protein NS5 ensures efficient RNA synthesis initiation and elongation by the polymerase domain. *Nucleic Acids Res*. **42** (2014). 10.1093/nar/gku666 PMC4191377 25209234

[15] Lim SP (2013). A crystal structure of the dengue virus non-structural protein 5 (NS5) polymerase delineates interdomain amino acid residues that enhance its thermostability and de novo initiationactivities. J. Biol. Chem..

[16] Zhao Yongqian, Soh Tingjin Sherryl, Zheng Jie, Chan Kitti Wing Ki, Phoo Wint Wint, Lee Chin Chin, Tay Moon Y. F., Swaminathan Kunchithapadam, Cornvik Tobias C., Lim Siew Pheng, Shi Pei-Yong, Lescar Julien, Vasudevan Subhash G., Luo Dahai (2015). A Crystal Structure of the Dengue Virus NS5 Protein Reveals a Novel Inter-domain Interface Essential for Protein Flexibility and Virus Replication. PLOS Pathogens.

[17] Selisko B (2006). Comparative mechanistic studies of de novo RNA synthesis by flavivirus RNA-dependent RNA polymerases. Virology.

[18] Zhao, B. *et al*. Structure and function of the Zika virus full-length NS5 protein. *Nat*. *Commun*. **8** (2017). 10.1038/ncomms14762 PMC5378950 28345656

[19] Wang Boxiao, Tan Xiao-Feng, Thurmond Stephanie, Zhang Zhi-Min, Lin Asher, Hai Rong, Song Jikui (2017). The structure of Zika virus NS5 reveals a conserved domain conformation. Nature Communications.

[20] Duan W (2017). The crystal structure of Zika virus NS5 reveals conserved drug targets. EMBO J..

[21] Upadhyay AK (2017). Crystal structure of full-length Zika virus NS5 protein reveals a conformation similar to Japanese encephalitis virus NS5. Acta Crystallogr. Sect. Struct. Biol. Commun..

[22] Lu Guoliang, Gong Peng (2013). Crystal Structure of the Full-Length Japanese Encephalitis Virus NS5 Reveals a Conserved Methyltransferase-Polymerase Interface. PLoS Pathogens.

[23] Maag David, Castro Christian, Hong Zhi, Cameron Craig E. (2001). Hepatitis C Virus RNA-dependent RNA Polymerase (NS5B) as a Mediator of the Antiviral Activity of Ribavirin. Journal of Biological Chemistry.

[24] Jin Z, Deval J, Johnson KA, Swinney DC (2011). Characterization of the elongation complex of dengue virus RNA polymerase: Assembly, kinetics of nucleotide incorporation, and fidelity. J. Biol. Chem..

[25] Appleby TC (2015). Structural basis for RNA replication by the hepatitis C virus polymerase. Science (80-.)..

[26] Pomerantz RT, Temiakov D, Anikin M, Vassylyev DG, McAllister WT (2006). A Mechanism of Nucleotide Misincorporation during Transcription due to Template-Strand Misalignment. Mol. Cell.

[27] Kaiyu M, Temiakov D, Jiang M, Anikin M, McAllister WT (2002). Major conformational changes occur during the transition from an initiation complex to an elongation complex by T7 RNA polymerase. J. Biol. Chem..

[28] Zuo Y, Steitz TA (2015). Crystal Structures of the *E. coli* Transcription Initiation Complexes with a Complete Bubble. Mol. Cell.

[29] Tahlrov TH (2002). Structure of a T7 RNA polymerase elongation complex at 2.9 Å resolution. Nature.

[30] Cheung ACM, Sainsbury S, Cramer P (2011). Structural basis of initial RNA polymerase II transcription. EMBO J..

[31] Bernecky C, Herzog F, Baumeister W, Plitzko JM, Cramer P (2016). Structure of transcribing mammalian RNA polymerase II. Nature.

[32] Korzheva N (2000). A structural model of transcription elongation. Science (80-.)..

[33] Potisopon S, Ferron F, Fattorini V, Selisko B, Canard B (2017). Substrate selectivity of Dengue and Zika virus NS5 polymerase towards 2′-modified nucleotide analogues. Antiviral Res..

[34] Šebera J (2018). The structural model of Zika virus RNA-dependent RNA polymerase in complex with RNA for rational design of novel nucleotide inhibitors. Sci. Rep..

[35] Calmels C, Ventura M, Aknin C, Métifiot M, Andreola ML (2017). *De novo* RNA synthesis catalyzed by the Zika Virus RNA polymerase domain. Sci. Rep..

[36] Jia Y, Patel SS (1997). Kinetic mechanism of transcription initiation by bacteriophage T7 RNA polymerase. Biochemistry.

[37] Godoy AS (2017). Crystal structure of Zika virus NS5 RNA-dependent RNA polymerase. Nat. Commun..

[38] Hercik K, Brynda J, Nencka R, Boura E (2017). Structural basis of Zika virus methyltransferase inhibition by sinefungin. Arch. Virol..

[39] Li Xiao-Dan, Shan Chao, Deng Cheng-Lin, Ye Han-Qing, Shi Pei-Yong, Yuan Zhi-Ming, Gong Peng, Zhang Bo (2014). The Interface between Methyltransferase and Polymerase of NS5 Is Essential for Flavivirus Replication. PLoS Neglected Tropical Diseases.

[40] Hercík K (2017). Adenosine triphosphate analogs can efficiently inhibit the Zika virus RNA-dependent RNA polymerase. Antiviral Res..

[41] Jain R, Butler KV, Coloma J, Jin J, Aggarwal AK (2017). Development of a S-adenosylmethionine analog that intrudes the RNA-cap binding site of Zika methyltransferase. Sci. Rep..

[42] Elfiky AA (2016). Zika viral polymerase inhibition using anti-HCV drugs both in market and under clinical trials. J. Med. Virol..

[43] Elfiky AA, Ismail AM (2018). Molecular docking revealed the binding of nucleotide/side inhibitors to Zika viral polymerase solved structures. SAR QSAR Environ. Res..

[44] Lu, G. *et al*. Analysis of ribonucleotide 5′-triphosphate analogs as potential inhibitors of Zika virus RNA-dependent RNA polymerase by using nonradioactive polymerase assays. *Antimicrob*. *Agents Chemother*. **61** (2017). 10.1128/AAC.01967-16 PMC5328514 27993851

[45] Kamiyama N (2017). Ribavirin inhibits Zika virus (ZIKV) replication *in vitro* and suppresses viremia in ZIKV-infected STAT1-deficient mice. Antiviral Res..

[46] Xu HT (2017). Purification of Zika virus RNA-dependent RNA polymerase and its use to identify small-molecule Zika inhibitors. J. Antimicrob. Chemother..

[47] Shan C (2016). An Infectious cDNA Clone of Zika Virus to Study Viral Virulence, Mosquito Transmission, and Antiviral Inhibitors. Cell Host Microbe.

