# Supplementary material for: Identification of a Small Interface between the Methyltransferase and RNA Polymerase of NS5 that is Essential for Zika Virus Replication
Source: Sci Rep. 2018 Nov 26;8:17384. doi: 10.1038/s41598-018-35511-3 (PMC6255901; doi:10.1038/s41598-018-35511-3)

Supplementary Figures

Identification of a Small Interface between the Methyltransferase and RNA Polymerase Domain of NS5 that is Essential for Zika Virus Replication

Authors/Affiliations:
Timur Rusanov[1], Tatiana Kent[1], Mohsan Saeed[2], Trung M. Hoang[1], Crystal Thomas[1], Charles M. Rice[2] and Richard T. Pomerantz[1]

Supplementary Figure 1 Comparison of strain-specific RdRp RNA synthesis activities.

a, Denaturing SDS gels showing purified RdRp encoded by Brazilian (SPH2015) and Asian (PRVABC59) strains. **b,** Schematic of RNA elongation assay (top). Denaturing gels showing a time course of single nucleotide incorporation by the indicated RdRp proteins. Template sequence indicated. (left). Plot showing % extension over time (right). **c,** Schematic of *de novo* RNA synthesis assay. Template sequence indicated. (left). Denaturing gel showing *de novo* synthesis performed by the indicated RdRp proteins in quadruplicate (middle). Bar chart showing relative intensities of *de novo* synthesis generated by the indicated RdRp proteins from middle gel. *p* value determined by non-paired, two-tailed student's *t*-test. Data represent mean, n = 4, ± s.d. RU = relative units. Uncropped and variable contrast gel images are presented in Supplementary Figure 6.

Supplementary Figure 2 Controls for *de novo* RNA synthesis.

a, Non-denaturing gel showing lack of RNA 3' terminal nucleotidyl transferase activity by NS5 in the presence of ATP. **b,** Schematic of initiation assay (left). Denaturing gel showing *de novo* synthesis for 30 min by NS5 wild-type and mutant proteins on indicated template with 10 µM NTPs (middle). Bar chart showing average *de novo* synthesis products generated after 30 min by NS5 wild-type and NS5 mutant enzymes. Data represent mean, n = 4, ± s.d. RU = relative units (right). Uncropped and variable contrast gel images are presented in Supplementary Figure 6.

Supplementary Figure 3

a, Uncropped scan of denaturing gel image of Figure 1d (left) and high-contrast scan of Figure 1d (right) in quadruplicate. **b,** Uncropped scan of denaturing gel image scan of Figure 1e (left) and high-contrast scan of Figure 1e in quadruplicate.

Supplementary Figure 4

a, Uncropped scan of denaturing gel image of Figure 2a (left) and high-contrast scan of Figure 2a. (right) in quadruplicate **b,** Uncropped scan of denaturing gel image scan of Figure 2b (left) and high-contrast scan of Figure 2b (right) in quadruplicate.

Supplementary Figure 5

a, Uncropped scan of denaturing gel image of Figure 3b. **b,** Uncropped scan of denaturing gel image of Figure 4c in triplicate. **c,** Uncropped scan of denaturing gel image scan of Supplementary Figure 2 in triplicate (left) and Figure 4d (right).

Supplementary Figure 6

a, Uncropped scan of denaturing gel image of Supplementary Figure 1b. **b,** Uncropped scan of denaturing gel image scan of Supplementary Figure 1c in quadruplicate.

Supplementary Figure 7

a. Denaturing gel showing time course of single nucleotide incorporation by Asian RdRp on the indicated template (left). Scatter plot showing % extension over time by RdRp (right). Data represent mean. n = 3, ± s.d. **b.** Denaturing SDS gels showing Asian NS5 and Asian RdRp after the indicated incubation period at 37° C in RNA elongation buffer lacking BSA, RNA template and NTPs.

Supplementary Fig. 1

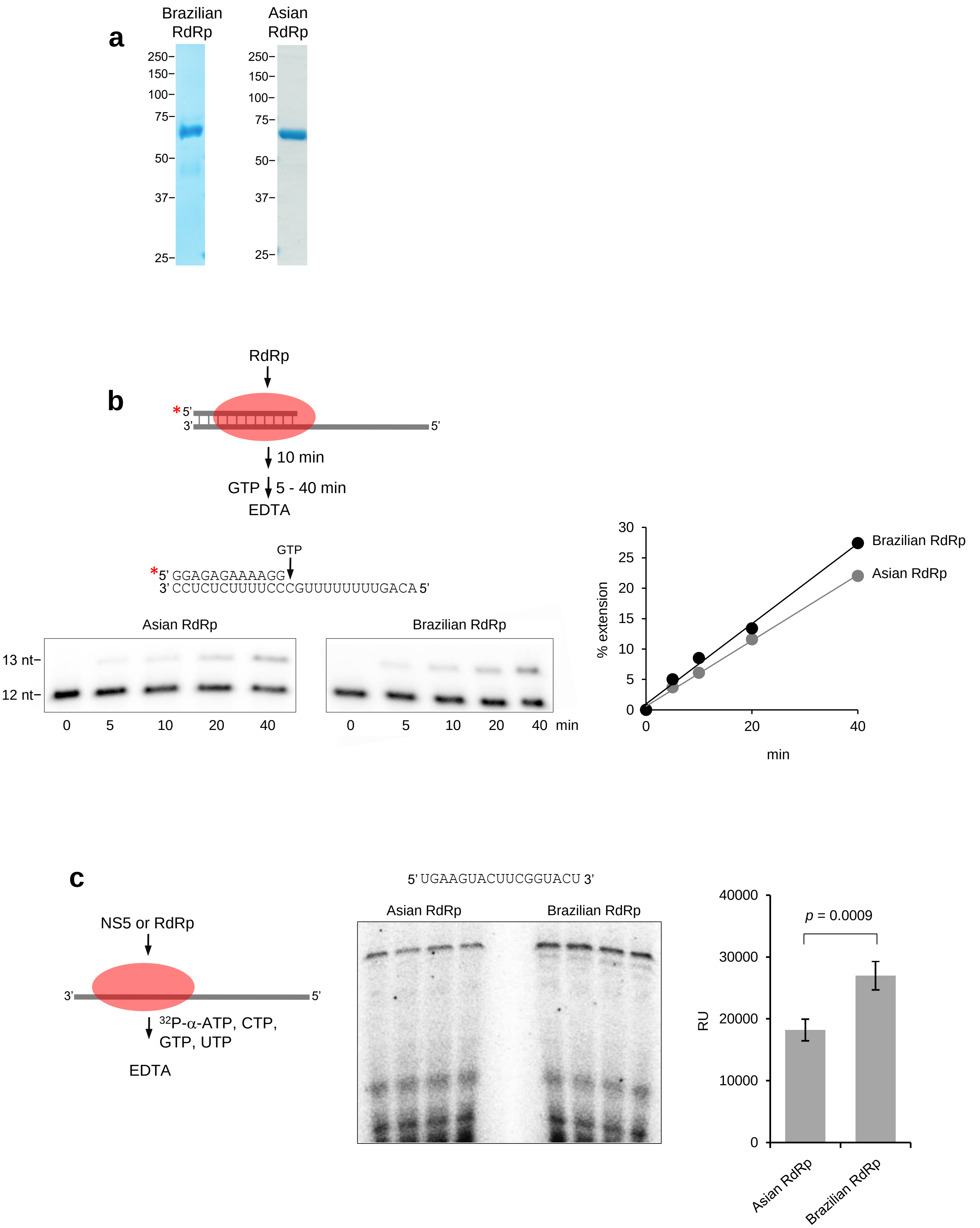

Supplementary Fig 2

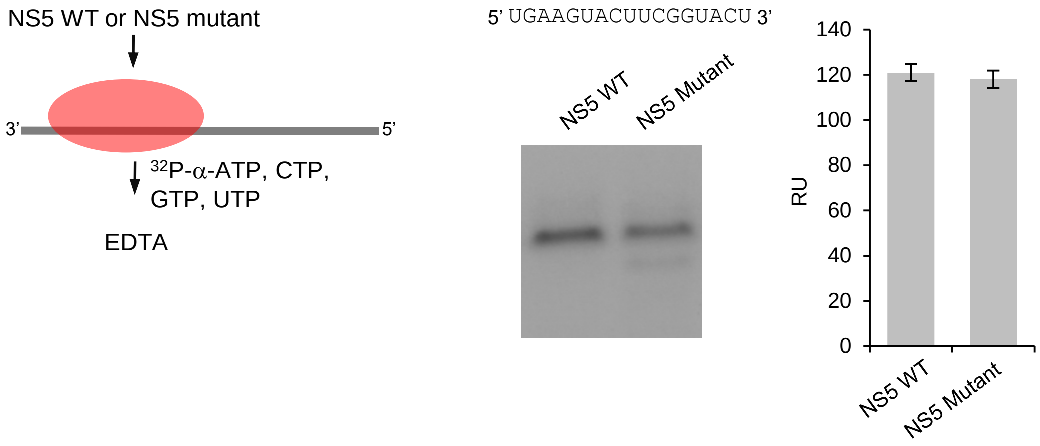

Supplementary Fig. 3

a

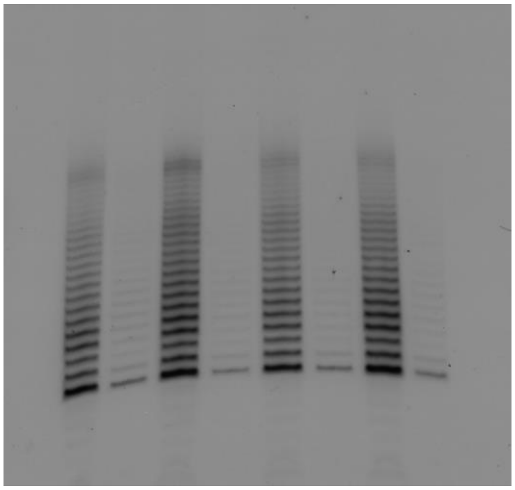

Full-length gel - Fig. 1d

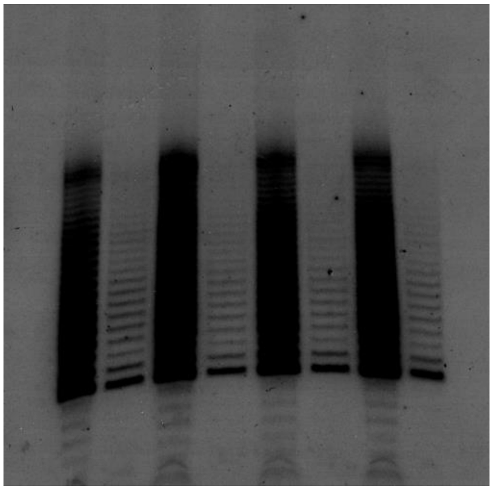

High contrast gel - Fig. 1d

b

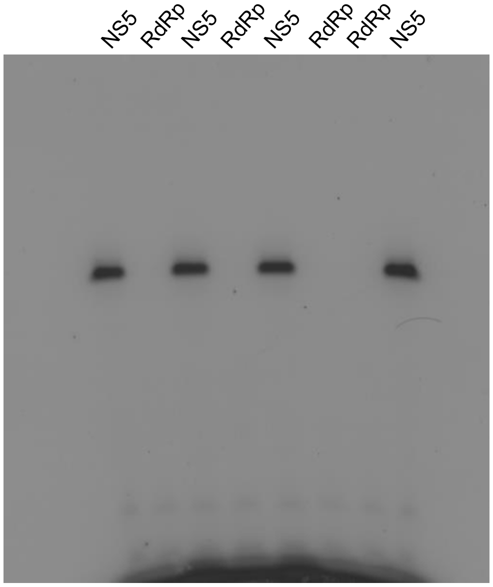

Full-length gel - Fig. 1e

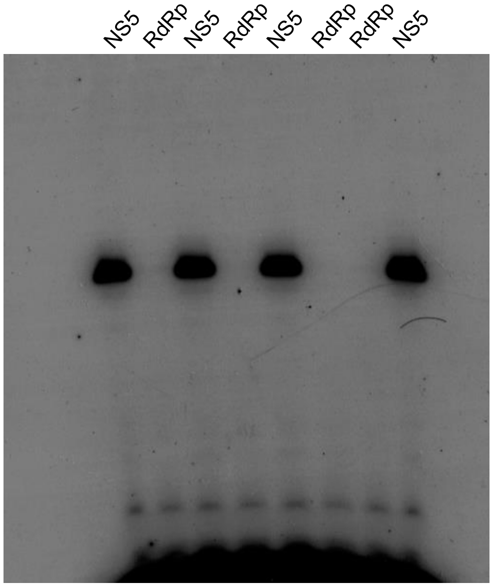

High contrast gel - Fig. 1e

Supplementary Fig. 4

a

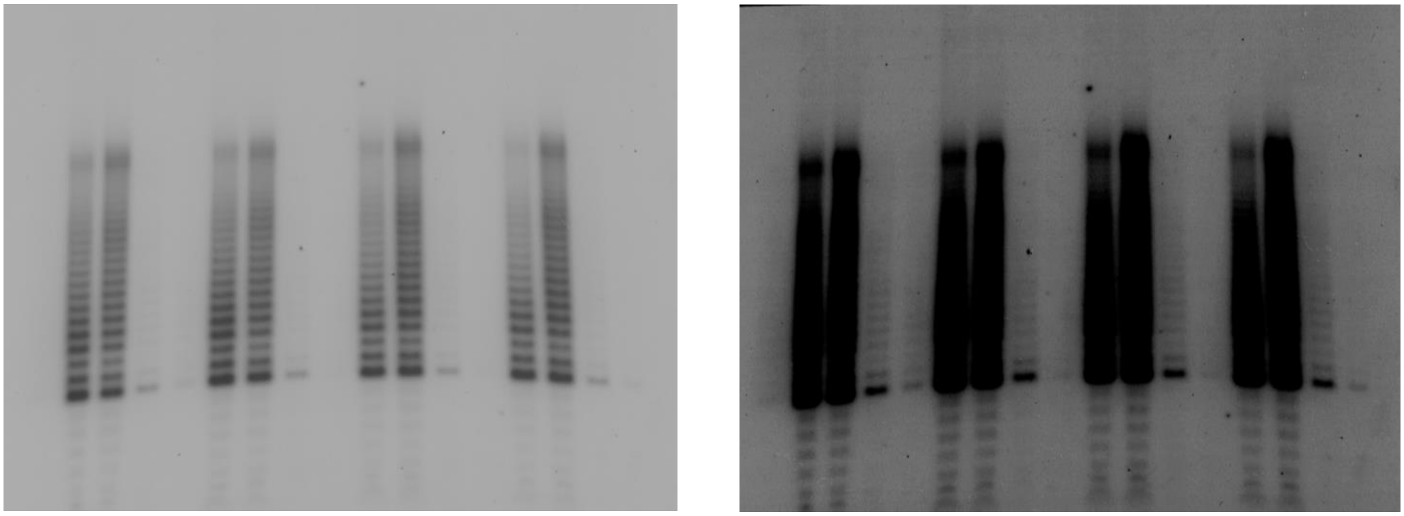

Full length gel - Fig. 2a

High contrast gel - Fig. 2a

b

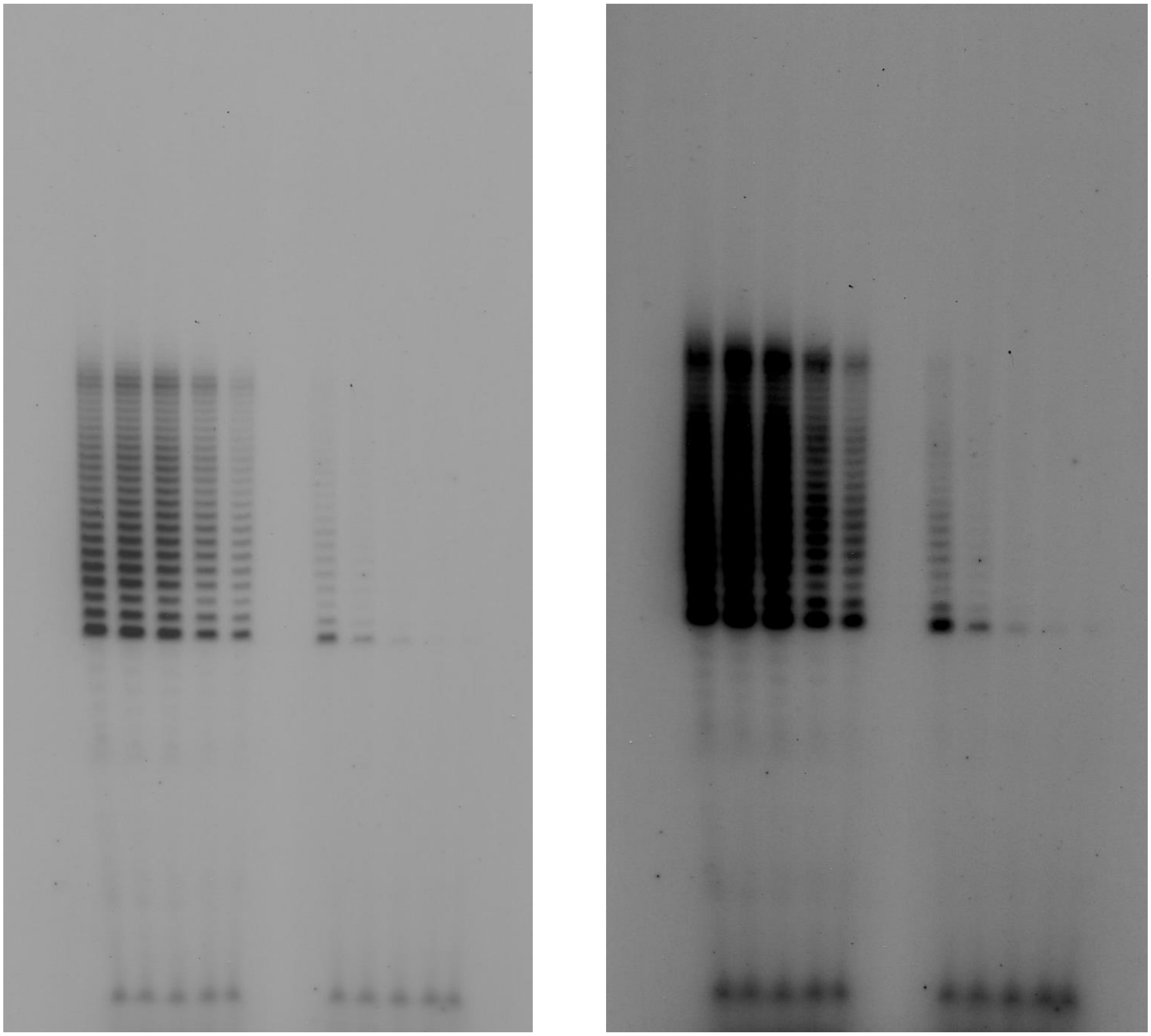

Full length gel - Fig. 2b

High contrast gel - Fig. 2b

Supplementary Fig. 5

a

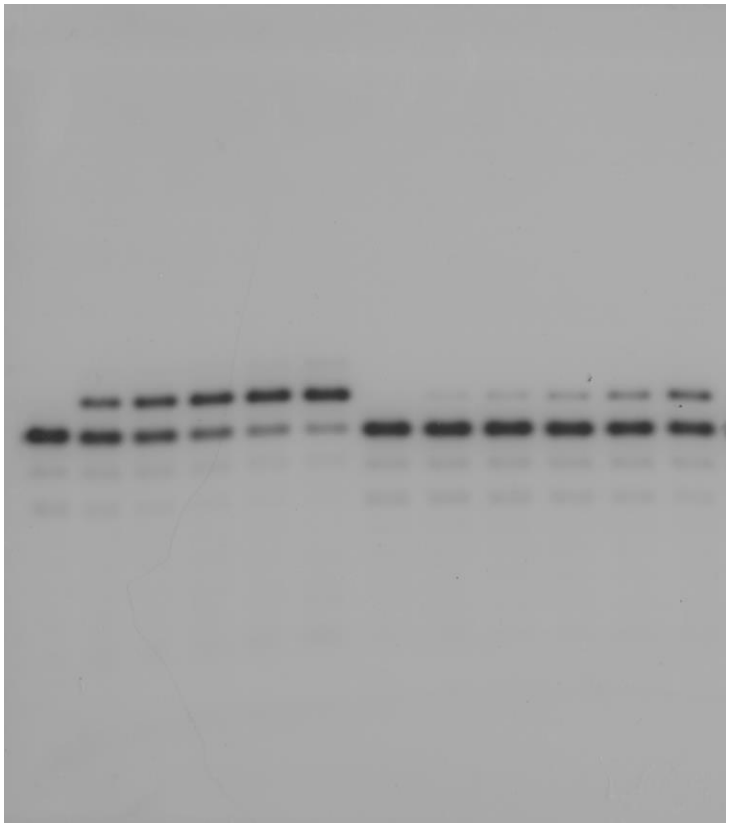

Full length gel - Fig. 3b

b

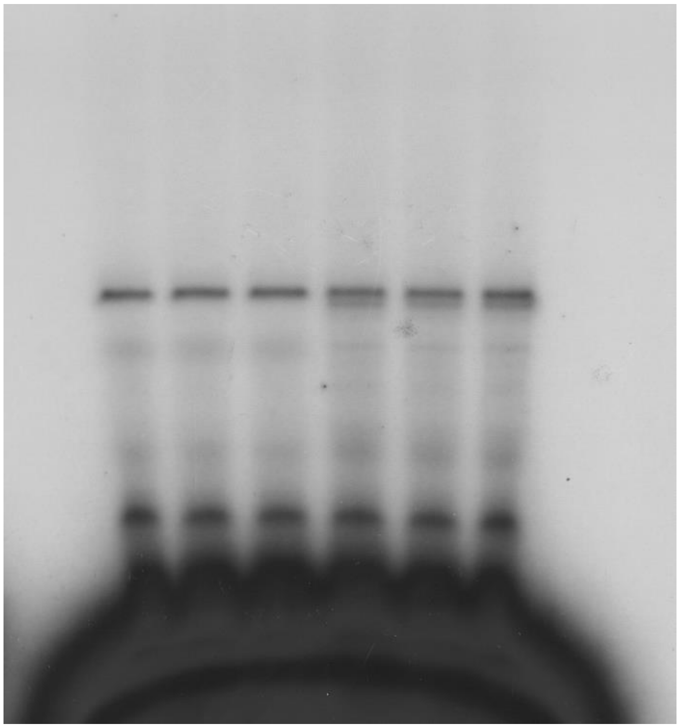

Full length gel – Fig. 4c

c

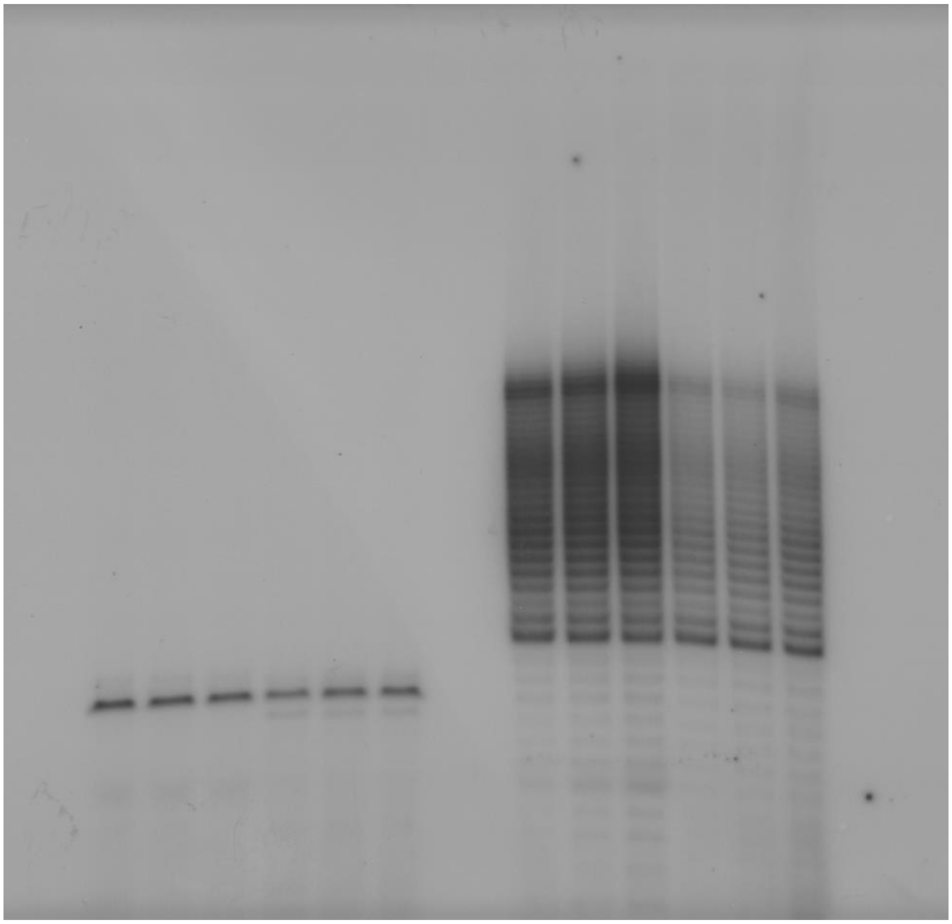

Full length gel – Supplementary Fig. 2 Full length gel – Fig. 4d

Supplementary Fig. 6

a

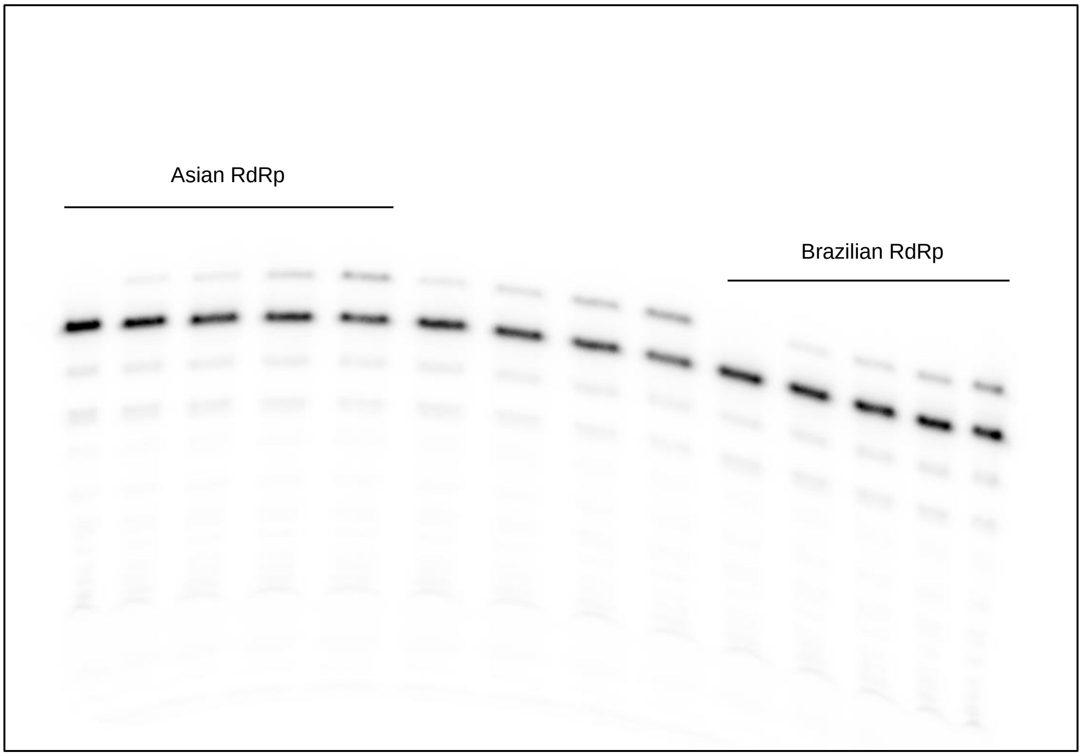

Full length gel – Supplementary Fig. 1b

b

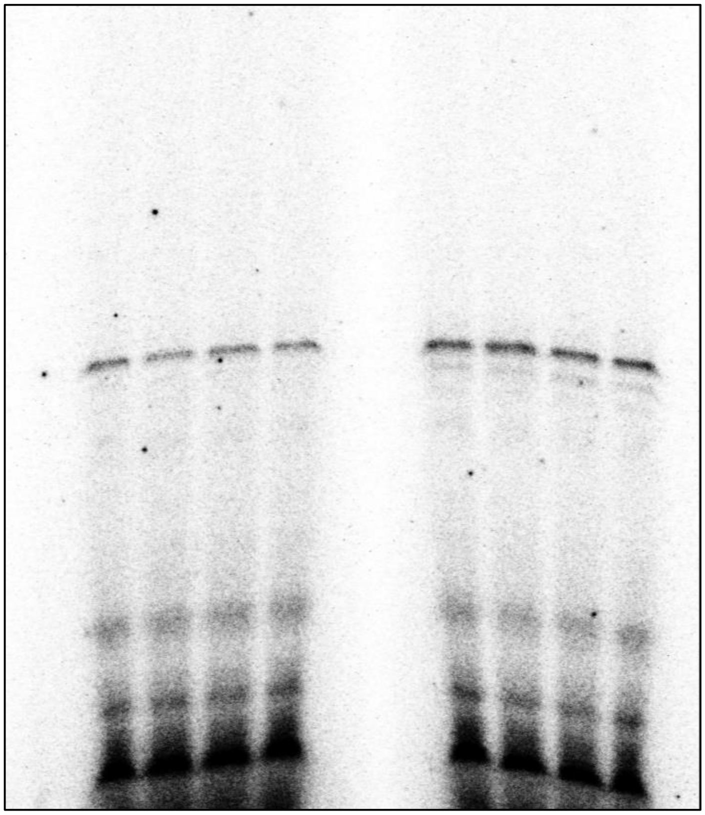

Full length gel – Supplementary Fig. 1c

Supplementary Fig. 7

a

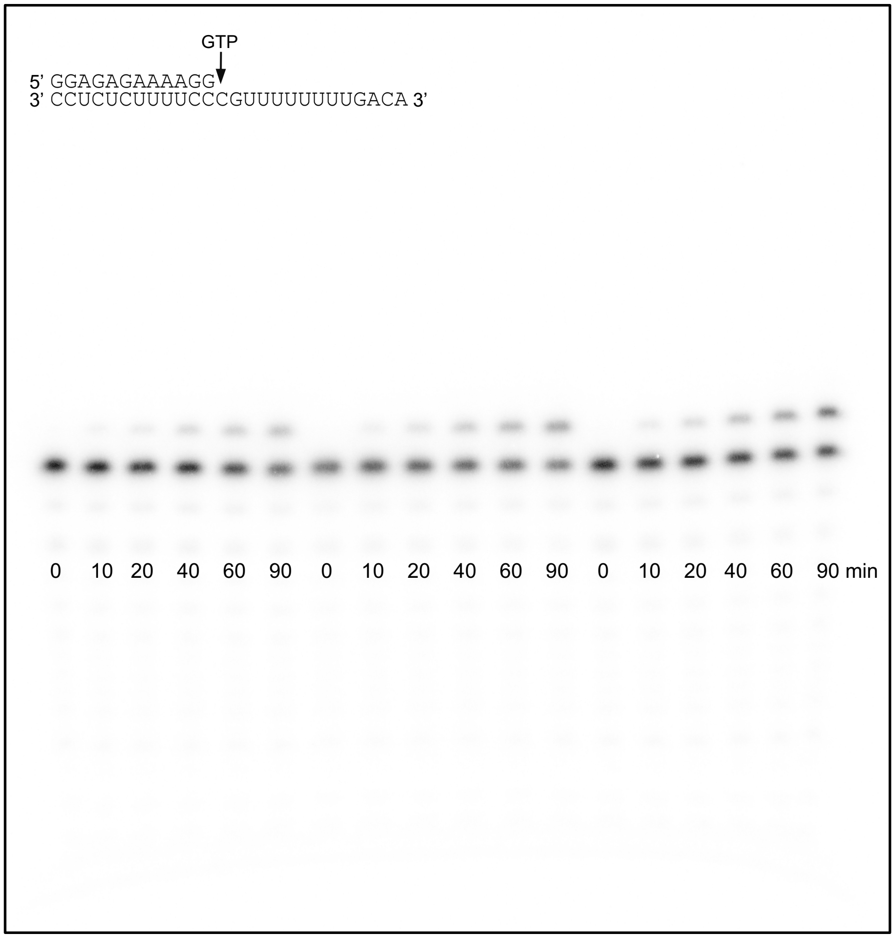

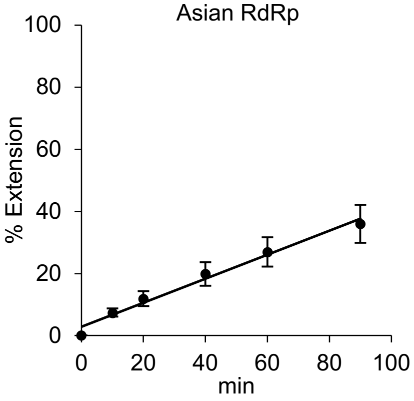

b

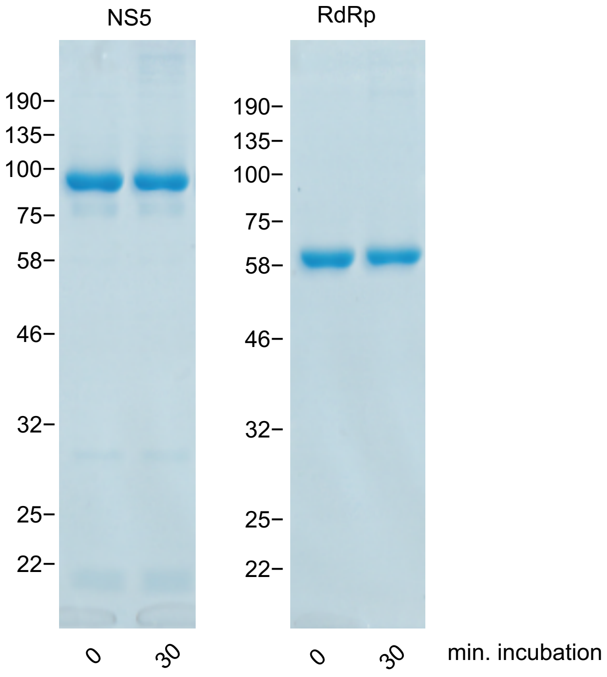

Supplement: Supplementary file 1 — Supplementary Figures [file 41598_2018_35511_MOESM1_ESM.pdf]
